# Talus Osteonecrosis after Chemotherapy: A Case Report

**DOI:** 10.1055/s-0043-1771004

**Published:** 2024-04-22

**Authors:** Larissa Macedo Barros, Pedro Paulo Ribeiro Cortez, Emanuella Vaccarezza de Souza, Eugênio Cesar Mendes

**Affiliations:** 1 Serviço de Ortopedia e Traumatologia, Hospital das Clínicas Samuel Libânio (HCSL), UNIVÁS, Pouso Alegre, MG, Brasil; 2 Serviço de Oncologia Pediátrica, Hospital das Clínicas Samuel Libânio (HCSL), UNIVÁS, Pouso Alegre, MG, Brasil

**Keywords:** chemotherapy, orthopedic, osteonecrosis, talus, traumatology

## Abstract

This paper described a case of talus osteonecrosis in a 13-year-old female with a diagnosis of T-type acute lymphocytic leukemia, who underwent chemotherapy and treatment with glucocorticoids, attended at the Orthopedics and Traumatology Sector of our institution. After approximately six months of treatment, the patient began to complain of sporadic pain in her left ankle with progressive worsening. Bone scintigraphy and magnetic resonance imaging of the ankles showed the presence of avascular osteonecrosis of the bilateral talar body. We opted for non-surgical treatment with analgesia and anti-inflammatory drugs, in addition to removal of the load associated with the use of immobilization of the extramedullary tutor for four weeks, followed by physical therapy rehabilitation with analgesia and progressive increase in load.

## Introduction

One of the main causes of atraumatic osteonecrosis is the use of glucocorticoids. These drugs induce osteoblast and osteocyte apoptosis, potentially impairing bone turnover. Corticosteroids potentiate osteoclast survival, which combined with their pro-apoptotic effect on osteoblasts, lead to a rapid decrease in bone mineral density. There are mechanisms by which glucocorticoids may also promote vascular compromise and ischemic necrosis by interfering with angiogenesis and the production and action of vascular endothelial growth factor. Furthermore, as osteocytes and osteoblasts promote angiogenesis, their apoptotic losses further decrease vascular growth. 1 2

## Case Report

This case report was approved by the ethics committee under number CAAE: 56114122.4.0000.5102.

Patient, female, ten years old, sought the pediatric oncology service complaining of the appearance of lymph nodes in the right cervical region, with progressive increase for approximately 14 days, associated with mild fatigue. She had no fever, night sweats, weight loss or other alterations.

At the first appointment, in July 2009, the patient presented enlarged anterior cervical, infrauricular, right submandibular, left clavicular and right inguinal fossa on physical examination. The largest cluster measured 10 cm in the right lateral cervical region. Bone marrow and imaging exams were performed, showing Acute Lymphoid Leukemia (ALL), type T, and treatment was then initiated based on the German BFM 2009 protocol on July 26, 2019, with good response.

**Fig. 1:**
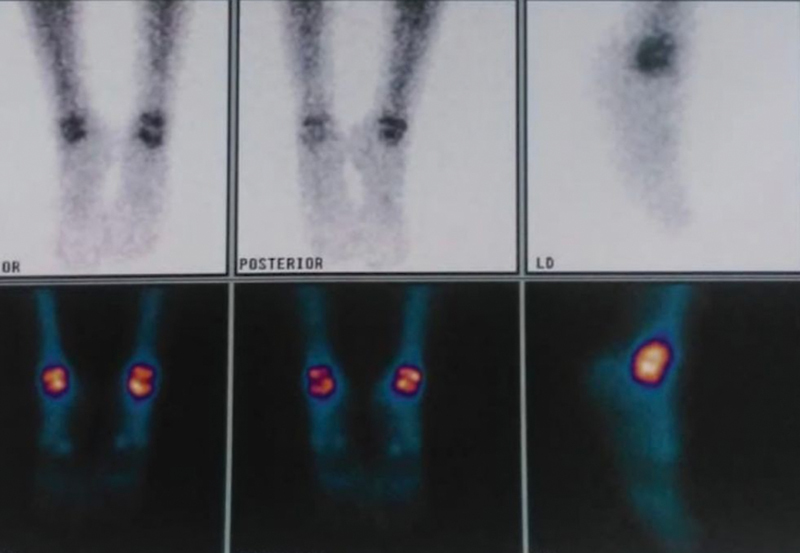
Ankle bone scintigraphy. Source: Service collection.

In December 2019, she started experiencing sporadic pain in her left ankle, with progressive worsening. Pain propaedeutics were then performed, and magnetic resonance imaging of the ankle was requested, in addition to bone scintigraphy. Imaging tests ( Figs. 1 e 3 ) showed avascular necrosis of the body of the talus, and temporary immobilization with a robofoot boot was indicated, in addition to load removal, followed by serial radiographs.

**Fig. 2:**
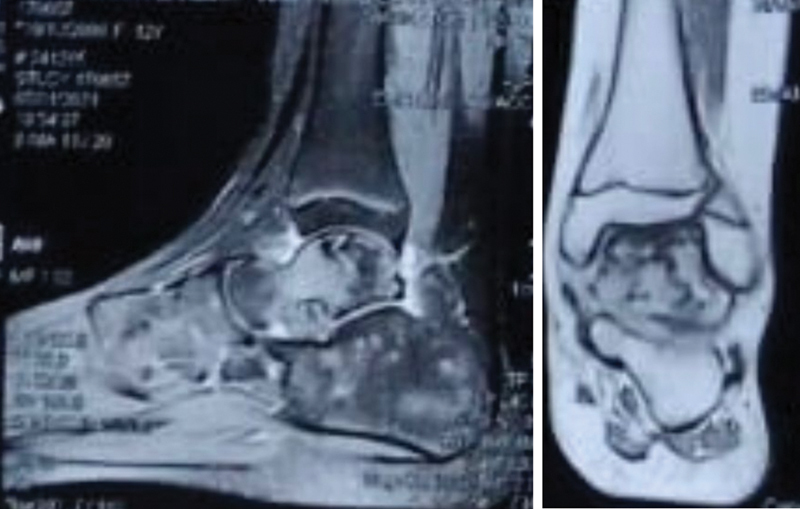
Magnetic resonance imaging of the ankle. Source: Service collection.

**Fig. 3:**
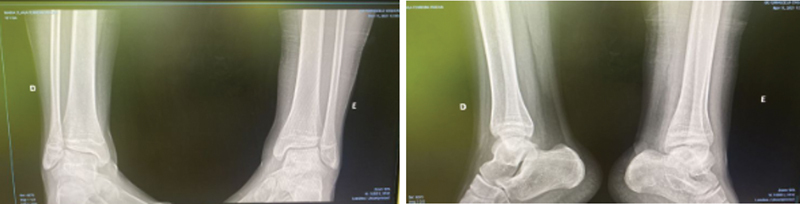
Ankle X-ray. Source: Service collection.

The patient evolved well, with progressive improvement in pain and active ankle movements. The radiograph showed improvement in the appearance of necrosis and bone remodeling ( Fig. 3 ).

In November 2021, there was a significant improvement in the clinical picture, with no complaints of pain and no claudication. The radiography showed bone remodeling, and the immobilization was removed. The patient was referred to physical therapy for rehabilitation, with gait training and progressive partial load, muscle strengthening and range of motion gain. In the following months, the patient returned for clinical evaluation, without pain complaints or any other symptomatology, being discharged from our Service, however, still being followed up by pediatric oncology.

## Discussion

Jones 3 described the development of biopsy-proven osteonecrosis at multiple sites, including bone, in a patient with bronchogenic small cell carcinoma who received short-term chemotherapy and corticosteroid administration. The author commented that multifocal osteonecrosis would have a wide variety of etiologies, but was most often found in the clinical setting of corticosteroid administration, connective tissue disorders, transplantation, haemoglobinopathies, and dysbarism. According to the researcher, in cancer patients, chemotherapy, the use of corticosteroids and bone marrow transplantation (with associated preparation therapy) were all implicated as possible causes, and there may be a synergistic effect when corticosteroids are used in combination with chemotherapy and radiotherapy. Multiple periarticular abnormalities appearing on serial radionuclide bone scintigraphy of the cancer patient, particularly when symmetrical and in a distribution not suggestive of metastatic bone disease, could raise the possibility of multifocal osteonecrosis. Multifocal infection and polysynovitis/arthritis of another etiology should also be considered in the differential diagnosis. Magnetic resonance imaging would have high sensitivity and specificity in the diagnosis of osteonecrosis and could be used when this condition is suspected. For the author, early diagnosis of osteonecrosis was important to prevent irreversible bone and joint destruction.

Bayram et al. 4 report the single case of an 11-year-old female patient with osteonecrosis of the left talus that developed six months after completion of the chemotherapy she received for ALL. According to the authors, a conservative treatment protocol was followed, including activity modification, analgesia, and prevention of weight bearing. However, the disease progressed significantly during the follow-up period.

According to Krull et al., 5 osteonecrosis is a common and debilitating side effect of antileukemic treatment in children with ALL. However, the impact of leukemia itself on the development of osteonecrosis still remained undefined. Thus, the authors analyzed 76 children with ALL, and magnetic resonance screening revealed 14 osteonecrotic lesions in this sample, 5 on the hips and 9 on the knees, which ranged from grades I to III in 9.2% of the patients. Six months after the first evaluation, an increase in the number of patients affected by osteonecrosis was observed, although with an increase in the degree of involvement in some patients, a reduction in others, in addition to stability and even resolution of the case. Furthermore, these results were not associated with age, pubertal stage, body mass index, leukemia characteristics or clinical presentation, suggesting that osteonecrosis may originate from the leukemic condition itself, and not just from the chemotherapy treatment.

Anyway, as the vast majority of children can be cured of ALL with chemotherapy and survive in the long term, assessing the morbidity and toxicity associated with current intensive treatment protocols for ALL in childhood becomes even more important. Therefore, it must be reaffirmed that osteonecrosis is one of the most common and debilitating side effects related to antileukemic therapy, and can adversely affect long-term quality of life. However, it is a known fact that the incidence and risk factors vary substantially between study groups and therapeutic regimens. For example, adolescent age is considered a significant risk factor for osteonecrosis in patients with ALL, especially those older than 10 years. Thus, a suitable approach to reduce the morbidity associated with the condition is early screening based on serial MRI images. 6

Still on the side effects of ALL treatment, corticosteroid-induced osteonecrosis is indeed a challenging complication, especially in childhood. The known risk factors, as mentioned, are age above 10 years, female gender and, especially, the use of dexamethasone. However, despite this fact, therapy with corticosteroids should not be suspended, 6 7 since their use is associated with improvement in ALL, which in itself is a condition with high morbidity and mortality.

The pathogenesis of osteonecrosis after chemotherapy and use of glucocorticoids is multifactorial and complex. It includes osteoblast suppression, osteocyte apoptosis, intramedullary lipocyte proliferation, and fatty hypertrophy, as well as adverse effects on nutrient arteries. These factors together contribute to the occurrence of thrombosis and fat embolism, causing damage to the endothelial and smooth muscle cells of the venous system, promoting more vascular stasis and ischemia. In addition, prothrombotic factors are increased in the blood, inducing alterations in the bone marrow, which begins to present a greasy appearance, with a risk of microembolism and a negative effect on microcirculation, resulting in a bone compartment syndrome. 8

The treatment of patients with chemotherapy-associated osteonecrosis is challenging for both the hematologist and the orthopedic surgeon. There are no evidence-based standardized therapeutic concepts or specially defined treatment guidelines for children and adolescents, while surgical or conservative treatment options are controversial. 8 One form of treatment for osteonecrosis resulting from chemotherapy for AAL is the use of zoledronic acid, which is well tolerated and improves joint pain in most patients. 9 It should be noted that zoledronic acid can prevent osteonecrosis if started with chemotherapy, but does not show benefits when administered late. Furthermore, it should be considered that its use may reduce the antileukemic efficacy of chemotherapy for ALL. 10

Another form of treatment for osteonecrosis associated with chemotherapy is the use of the vasoactive and stable prostacyclin analogue Iloprost, indicated in cases of systemic sclerosis and bone pain due to sickle cell crisis, among others. The application of the drug for osteonecrosis represents an off-label use of the drug, although it has shown promising results. However, like zoledronic acid, it must be applied at the beginning of chemotherapy so that it can improve pain and joint function, not demonstrating satisfactory results when used late. 8

Corticosteroid-associated osteonecrosis within a chemotherapy regimen is usually associated with coagulopathies, apoptosis abnormalities, and lipid metabolism dysfunction. Defining the exact dose of these drugs so that osteonecrosis does not develop is quite challenging, as there are a number of factors that determine such risk, which should lead to caution when such treatments are instituted.

In addition, osteonecrosis during chemotherapy is seen in the treatment of various neoplasms with the aforementioned drugs. Its early diagnosis is important to prevent irreversible bone and joint destruction, in addition to being essential for differentiating osteonecrosis from multifocal infections and polysynovitis and/or arthritis of other etiologies. Magnetic resonance imaging has high sensitivity and specificity in the diagnosis of osteonecrosis, and should be used when this condition is suspected. In addition, radiographs and scintigraphy are of great value for observing the progress of the problem.

When correctly diagnosed and at its beginning, non-surgical treatment of osteonecrosis caused by corticosteroids can be performed, as reported in the case described in this paper, with analgesia and anti-inflammatory drugs, in addition to the removal of the load associated with the use of extramedullary tutor-type immobilization followed by physiotherapy rehabilitation with analgesia and progressive load increase.
