# Supplementary material for: Talus Osteonecrosis after Chemotherapy: A Case Report
Source: Rev Bras Ortop (Sao Paulo). 2024 Apr 22;59(Suppl 2):e224–7. doi: 10.1055/s-0043-1771004 (PMC11679636; doi:10.1055/s-0043-1771004)
Supplement: Related texts

# Research article: Osteonecrose do tálus pós-quimioterapia: Relato de caso

**Keywords:** ortopedia, osteonecrose, quimioterapia, tálus, traumatologia

## Abstract

Este trabalho descreveu um caso de osteonecrose do tálus em um adolescente de 13 anos do sexo feminino com diagnóstico de leucemia linfocítica aguda do tipo T, submetido à quimioterapia e tratamento com glicocorticoides, atendido no Setor de Ortopedia e Traumatologia da nossa instituição. Após aproximadamente seis meses de tratamento, a paciente começou a queixar-se de dores esporádicas no tornozelo esquerdo com piora progressiva. A cintilografia óssea e ressonância magnética dos tornozelos evidenciaram a presença de osteonecrose avascular do corpo do tálus bilateral. Optou-se pelo tratamento não cirúrgico com analgesia e anti-inflamatórios, além de retirada da carga associada à utilização de imobilização do tipo tutor extramedular por quatro semanas, seguida por reabilitação fisioterápica com analgesia e aumento progressivo da carga.

## Introdução

Uma das principais causas da osteonecrose atraumática é a utilização glicocorticoides. Estes medicamentos induzem a apoptose de osteoblastos e osteócitos, prejudicando potencialmente a remodelação óssea. Os corticosteroides potencializam a sobrevivência dos osteoclastos, que combinado com seu efeito pró-apoptótico nos osteoblastos, para levar a uma rápida diminuição da densidade mineral óssea. Existem mecanismos pelos quais os glicocorticoides também podem promover comprometimento vascular e necrose isquêmica, interferindo na angiogênese e na produção e ação do fator de crescimento endotelial vascular. Além disso, como os osteócitos e osteoblastos promovem a angiogênese, suas perdas apoptóticas diminuem ainda mais o crescimento vascular. 1 2

## Relato do Caso

Este relato de caso teve a aprovação do comitê de ética sob o número CAAE: 56114122.4.0000.5102.

Paciente, sexo feminino, dez anos, procurou o serviço de oncologia pediátrica com queixa de surgimento de linfonodos em região cervical à direita, com aumento progressivo há aproximadamente 14 dias, associada à fadiga leve. Negava febre, sudorese noturna, perda de peso ou outras alterações.

Na primeira consulta, em julho de 2009, a paciente apresentava ao exame físico linfonodomegalias cervicais anteriores, infrauriculares, submandibular direita, fossa clavicular esquerda e inguinal direita. O maior conglomerado media 10 cm em região cervical lateral direita. Foram realizados exames de medula e de imagem, evidenciando Leucemia Linfoide Aguda (LLA), tipo T, sendo então iniciado tratamento com base no protocolo alemão BFM 2009 em 26 de julho de 2019, com boa resposta.

Em dezembro de 2019 iniciou com dores esporádicas em tornozelo esquerdo, com piora progressiva. Foi realizada então propedêutica de dor, e solicitada ressonância nuclear magnética do tornozelo, além de cintilografia óssea. Os exames de imagem ( Figs. 1 e 3 ) evidenciaram necrose avascular do corpo do tálus, sendo indicada imobilização temporária com bota do tipo *robofoot* , além de retirada de carga, seguida de radiografias seriadas.

**Fig. 1:** Cintilografia óssea do tornozelo. Fonte: Acervo do Serviço.

**Fig. 2:** Ressonância nuclear magnética do tornozelo. Fonte: Acervo do Serviço.

**Fig. 3:** Radiografia do tornozelo. Fonte: Acervo do Serviço.

A paciente apresentou boa evolução, com melhora progressiva da dor e dos movimentos ativos do tornozelo. A radiografia evidenciou melhora do aspecto de necrose e remodelação óssea ( Fig. 3 ).

Em novembro de 2021, houve melhora significativa do quadro clínico, sem queixas álgicas e sem claudicação. A radiografia evidenciou remodelação óssea, sendo optado por retirada da imobilização. A paciente foi encaminhada à fisioterapia para reabilitação, com treino de marcha e carga parcial progressiva, fortalecimento muscular e ganho de amplitude de movimento. Nos meses subsequentes a paciente retornou para avaliação clínica, sem queixas álgicas ou qualquer outra sintomatologia, recebendo alta do nosso Serviço, porém, ainda em acompanhamento com a oncologia pediátrica.

## Discussão

Jones, 3 descreveu o desenvolvimento de osteonecrose em múltiplos sítios, inclusive óssea, comprovada por biópsia, em um paciente com carcinoma broncogênico de pequenas células que recebeu quimioterapia e administração de corticosteroide a curto prazo. O autor comentou que a osteonecrose multifocal teria uma ampla variedade de etiologias, mas era mais frequentemente encontrada no cenário clínico de administração de corticosteroides, distúrbios do tecido conjuntivo, transplante, hemoglobinopatias e disbarismo. Segundo o pesquisador, no paciente oncológico, a quimioterapia, o uso de corticosteroides e o transplante de medula óssea (com terapia de preparação associada) foram todos implicados como possíveis causas, podendo haver um efeito sinérgico quando os corticosteroides são utilizados em combinação com quimioterapia e radioterapia. Múltiplas anormalidades periarticulares que aparecem na cintilografia óssea seriada com radionuclídeos do paciente com câncer, particularmente quando simétricas e em uma distribuição não sugestiva de doença metastática óssea, poderiam levantar a possibilidade de osteonecrose multifocal. Também devem ser considerados no diagnóstico diferencial a infecção multifocal e a polissinovite / artrite de outra etiologia. A ressonância magnética teria alta sensibilidade e especificidade no diagnóstico de osteonecrose e poderia ser utilizada quando houver suspeita dessa condição. Para o autor, o diagnóstico precoce da osteonecrose era importante para prevenir a destruição óssea e articular irreversível.

Bayram et al. 4 relataram o caso único de uma paciente do sexo feminino de 11 anos de idade com osteonecrose no tálus esquerdo que se desenvolveu seis meses após o término da quimioterapia que recebeu para LLA. Segundo os autores, um protocolo de tratamento conservador foi seguido, incluindo modificação da atividade, analgesia e prevenção de descarga de peso. No entanto, a doença progrediu significativamente durante o período de acompanhamento.

Segundo Krull et al., 5 a osteonecrose é um efeito colateral comum e debilitante do tratamento antileucêmico em crianças com LLA. No entanto, o impacto da própria leucemia no desenvolvimento da osteonecrose ainda permanecia indefinido. Sendo assim, os autores analisaram 76 crianças com LLA, e a triagem por ressonância magnética revelou 14 lesões osteonecróticas nessa amostra, sendo 5 nos quadris e 9 nos joelhos, que variaram dos graus I ao III em 9,2% dos pacientes. Passados seis meses da primeira avaliação, foi observado aumento no número de pacientes acometidos pela osteonecrose, embora com aumento do grau de acometimento em alguns pacientes, redução em outros, além de estabilidade e até resolução do caso. Ainda, estes resultados não estiveram associados com idade, estágio puberal, índice de massa corporal, características da leucemia ou apresentação clínica, sugerindo que a osteonecrose pode ser oriunda da própria condição leucêmica, e não somente do tratamento quimioterápico.

De qualquer forma, como a grande maioria das crianças pode ser curada da LLA com quimioterapia e sobreviver em longo prazo, avaliar a morbidade e toxicidade associadas aos atuais protocolos de tratamento intensivo da LLA na infância torna-se ainda mais importante. Sendo assim, deve-se reafirmar que a osteonecrose é um dos efeitos colaterais mais comuns e debilitantes relacionados à terapia antileucêmica, e pode afetar adversamente a qualidade de vida em longo prazo. Todavia, é fato conhecido que a incidência e os fatores de risco variam substancialmente entre os grupos de estudo e regimes terapêuticos. Por exemplo, a idade adolescente é considerada um fator de risco significativo para osteonecrose em portadores de LLA, especialmente aqueles com idades maiores do que 10 anos. Assim, uma abordagem adequada para reduzir a morbidade associada à condição é a triagem precoce com base em imagens seriadas de ressonância magnética. 6

Ainda sobre os efeitos colaterais do tratamento da LAA, a osteonecrose induzida por corticosteroides é realmente uma complicação desafiadora, especialmente na infância. Os fatores de risco conhecidos, como dito, são a idade acima de 10 anos, além de sexo feminino e, especialmente, o uso de dexametasona. Porém, a despeito deste fato, a terapia com os corticosteroides não deve ser suspensa, 6 7 uma vez que sua utilização está associada à melhora da LLA, que por si só é uma condição de elevada morbimortalidade.

A patogênese da osteonecrose após a quimioterapia e uso de glicocorticoides é multifatorial e complexa. Inclui a supressão de osteoblastos, apoptose de osteócitos, proliferação de lipócitos intramedulares e hipertrofia gordurosa, além de efeitos adversos nas artérias nutrícias. Estes fatores em conjunto contribuem para a ocorrência de trombose e embolia gordurosa, gerando danos às células endoteliais e musculares lisas do sistema venoso, promovendo mais estase vascular e isquemia. Além disso, os fatores pró-trombóticos estão aumentados no sangue, induzindo alterações da medula óssea que passa a apresentar um aspecto gorduroso, com risco de microembolia e efeito negativo na microcirculação, resultando em uma síndrome compartimental óssea. 8

O tratamento de pacientes com osteonecrose associada à quimioterapia é desafiador tanto para o hematologista quanto para o cirurgião ortopédico. Não existem conceitos terapêuticos padronizados baseados em evidências ou diretrizes de tratamento especialmente definidos para crianças e adolescentes, enquanto opções de tratamento cirúrgico ou conservador são controversas. 8 Uma forma de tratamento da osteonecrose decorrente de quimioterapia para a LAA é a utilização de ácido zoledrônico, que é bem tolerado e melhora a dor articular na maioria dos pacientes. 9 Cabe ressaltar que o ácido zoledrônico pode prevenir a osteonecrose se iniciado com a quimioterapia, mas não mostra benefícios quando administrado tardiamente. Ainda, deve-se considerar que a sua utilização pode reduzir a eficácia antileucêmica da quimioterapia para LLA. 10

Outra forma de tratamento da osteonecrose associada à quimioterapia é a utilização do análogo de prostaciclina vasoativo e estável Iloprost, indicado nos casos de esclerose sistêmica e dor óssea por crise falciforme, dentre outras. A aplicação do medicamento para a osteonecrose representa um uso *off-label* da droga, embora tenha demonstrado resultados promissores. Porém, assim como o ácido zoledrônico, deve ser aplicado no início da quimioterapia para que possa melhorar a dor e a função articular, não demonstrando resultados satisfatórios quando utilizado tardiamente. 8

A osteonecrose associada à corticosteroides dentro de um esquema quimioterápico está associada usualmente a coagulopatias, anormalidades na apoptose e disfunção do metabolismo lipídico. A definição da dose exata destes fármacos de forma que a osteonecrose não se desenvolva é bastante desafiadora, pois existem uma série de fatores que determinam tal risco, o que deve gerar cautela quando tais tratamentos forem instituídos.

Além disso, a osteonecrose durante a quimioterapia é vista no tratamento de diversas neoplasias com os medicamentos supracitados. Seu diagnóstico precoce é importante para prevenir a destruição óssea e articular irreversível, além de essencial para a diferenciação da osteonecrose de infecções multifocais e a polissinovites e/ou artrites de outras etiologias. A ressonância magnética apresenta alta sensibilidade e especificidade no diagnóstico de osteonecrose, e deve ser utilizada quando houver suspeita dessa condição. Além disso, radiografias e cintilografias são de grande valia para a observação do avanço da problemática.

Quando diagnosticada corretamente e em seu início, o tratamento não cirúrgico da osteonecrose provocada por corticosteroides pode ser realizado, como relatado no caso descrito neste trabalho, com analgesia e anti-inflamatórios, além da retirada da carga associada à utilização de imobilização do tipo tutor extramedular seguida por reabilitação fisioterápica com analgesia e aumento progressivo da carga.
